# Principles of Molecular Utility for CMS Classification in Colorectal Cancer Management

**DOI:** 10.3390/cancers15102746

**Published:** 2023-05-13

**Authors:** Leili Rejali, Romina Seifollahi Asl, Fatemeh Sanjabi, Nayeralsadat Fatemi, Hamid Asadzadeh Aghdaei, Mahsa Saeedi Niasar, Pardis Ketabi Moghadam, Ehsan Nazemalhosseini Mojarad, Enrico Mini, Stefania Nobili

**Affiliations:** 1 Basic and Molecular Epidemiology of Gastrointestinal Disorders Research Center, Research Institute for Gastroenterology and Liver Diseases, Shahid Beheshti University of Medical Sciences, Tehran P.O. Box 19875-17411, Iran; leilirejali@gmail.com (L.R.); romina.seif76@gmail.com (R.S.A.); n.fatemi@sbmu.ac.ir (N.F.); hamid.asadzadeh@sbmu.ac.ir (H.A.A.); mahsa85.saeedi@gmail.com (M.S.N.); ketabimoghadam.p@sbmu.ac.ir (P.K.M.); 2 Department of Medical Biotechnology, School of Allied Medicine, Iran University of Medical Sciences, Tehran P.O. Box 14496-14535, Iran; fa.sanjabi@gmail.com; 3 Gastroenterology and Liver Diseases Research Center, Research Institute for Gastroenterology and Liver Diseases, Shahid Beheshti University of Medical Sciences, Yaman Street, Chamran Expressway, Tehran P.O. Box 19857-17411, Iran; e.nazemalhosseini@sbmu.ac.ir; 4 Department of Health Sciences, University of Florence, Viale Pieraccini, 6, 50139 Firenze, Italy; enrico.mini@unifi.it; 5 Department of Neuroscience, Psychology, Drug Research and Child Health—NEUROFARBA—Pharmacology and Toxicology Section, University of Florence, Viale Pieraccini, 6, 50139 Firenze, Italy

**Keywords:** colorectal cancer, cancer risk, consensus molecular subtype, immunoscore, gut microbiota, biomarkers predictive of prognosis, biomarkers predictive of drug response, precision medicine

## Abstract

**Simple Summary:**

Colorectal cancer is the second most deadly tumor worldwide, despite the availability of screening plans and advanced treatment strategies. Molecular biomarkers predictive of prognosis have been identified, although there is currently limited knowledge of biomarkers predictive of the response to pharmacological treatment. In recent years, molecular classifications mainly able to predict colorectal cancer prognosis and recurrence risk have been proposed. The “consensus molecular subtype (CMS) classification” is the most known and has contributed to the understanding of genomic and epigenomic landscapes of colorectal cancer for better patient management. This classification categorizes colorectal cancer into four CMS categories (CMS1–4) that display different prognosis. This manuscript contextualized the CMS classification in different settings, discussing its relationships with precursor lesions, tumor immunophenotype, and gut microbiota, as well as its role in predicting prognosis and/or response to pharmacological treatments, as a crucial step towards precision medicine.

**Abstract:**

Colorectal cancer (CRC) is the second cause of cancer-related deaths in both sexes globally and presents different clinical outcomes that are described by a range of genomic and epigenomic alterations. Despite the advancements in CRC screening plans and treatment strategies, the prognosis of CRC is dismal. In the last two decades, molecular biomarkers predictive of prognosis have been identified in CRC, although biomarkers predictive of treatment response are only available for specific biological drugs used in stage IV CRC. Translational clinical trials mainly based on “omic” strategies allowed a better understanding of the biological heterogeneity of CRCs. These studies were able to classify CRCs into subtypes mainly related to prognosis, recurrence risk, and, to some extent, also to treatment response. Accordingly, the comprehensive molecular characterizations of CRCs, including The Cancer Genome Atlas (TCGA) and consensus molecular subtype (CMS) classifications, were presented to improve the comprehension of the genomic and epigenomic landscapes of CRCs for a better patient management. The CMS classification obtained by the CRC subtyping consortium categorizes CRC into four consensus molecular subtypes (CMS1–4) characterized by different prognoses. In this review, we discussed the CMS classification in different settings with a focus on its relationships with precursor lesions, tumor immunophenotype, and gut microbiota, as well as on its role in predicting prognosis and/or response to pharmacological treatments, as a crucial step towards precision medicine.

## 1. Introduction

Colorectal cancer (CRC) is one of the most prevalent cancers with a high mortality rate, and it is a crucial public health issue [1]. In 2021, GLOBOCAN statistics revealed that CRC is the third most common cancer after breast and lung cancers. Moreover, it is the second highest cause of cancer-related deaths in both sexes. Despite the advancements in the CRC screening strategies utilized in societies, the occurrence of this malignancy is growing. Surprisingly, the mortality incidence is up to two to three times higher in developed versus underdeveloped countries due to demographic changes, although this may be further exacerbated by increasing risk factors associated with globalization and a growing economy [2]. Additionally, incidence increment in young adults points to the influence of lifestyle-related factors, such as smoking, heavy alcohol consumption, unhealthy dietary patterns, and reduced physical activity, which cause excess body mass, as well as racial background, personal or familial history of inflammatory bowel disease, family history of CRC or colorectal polyps, and inherited syndromes [3].

Key driver events in CRC progression, such as *KRAS*, *BRAF*, and *PIK3CA* mutations, and MSI status have been established, and, along with TNM staging, help clinicians in the clinical management of CRC [4].

In the last few years, molecular signatures able to classify CRC in molecular subtypes have been shown to play a critical role in prognosis. Although they are still not recommended by NCCN treatment cancer guidelines [5], their application could be useful in the near future. Various high-throughput techniques useful to establish the molecular subtypes and based on specific and reliable multi-omics approaches are available.

In the current narrative review, we provided a detailed overview of the CRC staging systems from the classical to high-throughput classifications, and their clinical significance in CRC. Then, we focused on the potential use of CMS classification in colon precursor lesions, the correlation between the CMS stratification and gut microbiota, and predicting prognosis and/or response to the pharmacological treatment, as a crucial step towards precision medicine.

## 2. CRC Management Based on Staging Systems

The CRC staging system is based on the TNM (T—primary tumor, N—regional lymph nodes’ status, and M—distant metastases) staging system.

### 2.1. TNM Staging, a Pathological Evaluation of CRC

CRC is a molecularly heterogeneous malignancy with various clinical outcomes. This characteristic makes the CRC classification more challenging [6]. The management of CRC usually still basically relies on the tumor location, and the stage of disease is defined according to the American Joint Committee on Cancer (AJCC), with TNM staging (tumor, nodes, metastasis) as the main predictive factor of prognosis [7,8,9]. The TNM staging provides information about the prognosis of the patient’s disease as a post-surgical stratification based on the pathologist’s evaluation of the resected tumor, assists in decision making for the adjuvant treatment, if needed, and confirms the presence/absence of metastasis at the time of diagnosis. The main failing point of TNM classification is the inability to discriminate between “good” and “poor” cancer prognosis within the same stage. Inadequate prediction of the exact outcome of patients is concerning, particularly in stages II and III, because nearly 20% of patients defined in stage II may still die due to recurrence [10]. Patients detected within the same TNM stage may also experience different outcomes in relation to various genotypic and phenotypic differences that exist in CRC patients [11]. Accordingly, unnecessary treatment or over-treatment may occur through inaccurate discrimination of the disease. The TNM system, widely used in cancer staging, places considerable emphasis on the lymph node status. This aspect has been the subject of significant controversies, given the imprecise nature of the system. Consequently, researchers are actively exploring alternative or supplementary features that could enhance the precision of cancer staging [10]. Therefore, refinements of biological parameters to improve the stratification of patients for tumor treatment or surveillance strategies are under investigation.

### 2.2. Molecular Staging, Genetic and Epigenetic Characteristics of CRC

The molecular subtyping application of CRC as a heterogeneous disease is potentially beneficial [12]. Molecular classifications based on genetic and epigenetic characteristics in CRC patients rely on mutations, microsatellite instability (MSI), CpG island methylator phenotype (CIMP), chromosomal instability (CIN), copy-number deviations (SCNA), and significant pathways that affect CRC initiation and progression, such as WNT and MYC, which are employed for CRC stratification [8,13].

Differences in tumor biology are reflected by specific supervised expression signatures [11]. Several expression-based assays (e.g., Oncotype DX Colon, ColoPrint, and ColDx/GeneFX) with prognostic value for CRC are commercially available, but none of these signatures are yet validated and recommended for clinical use [5,14].

Developments in genomic, transcriptomic, and big-data technologies enable investigators to explore the molecular characteristics of tumors and define their clinical relevance. The integrative omics analyses reveal the potential of incorporating different biological levels in which the transcriptome may play a valuable role [14]. We discuss these items in more detail below.

(i) CRC development from benign to malignant lesions, induced by some key driver genes, acquires a series of mutations over time. Among the key driver genes that play role in carcinogenesis, the adenomatous polyposis coli (APC), accompanied by its mutations, regulates growth advantages in epithelial cells and results in the formation of a small adenoma. Later, the generation of mutations in *KRAS* and *BRAF* provides a second round of expansion for cells, involving transformation to a large adenoma. Finally, the occurrence of *PIK3CA*, *SMAD4*, and *p53* mutations develops a malignant tumor that has the potential for invasion and metastasis. It remains unclear which mutations of these key driver genes are involved in the metastasis of CRC [15].
(ii) MSI is caused by mutations in DNA mismatch repair genes (*MLH1*, *MSH2*, *MSH6*, and *PMS2*) and *EPCAM*. The occurrence of mutations in *MLH1* or *MSH2* genes leads to an increased risk (70–80%) of developing cancer, while mutations in the *MSH6* or *PMS2* genes have a comparatively lower risk (25–60%) of cancer development [16]. Nearly 15–20% of primary CRCs have the MSI phenotype, whereas the remainders are microsatellite stable (MSS). MSI tumors demonstrate multiple single nucleotide variants (SNVs) and insertion/deletions (indels). The five microsatellite markers were used as a standard panel to access the MSI status of cancer, including BAT26 and BAT25 as mononucleotide repeats, plus three dinucleotide repeats (D2S123, D5S346, and D17S250), as defined by the Bethesda Guidelines [17]. A subset of tumors with unstable loci in ≥30% markers are defined as “microsatellite high” (MSI-H), a subset of tumors with 10–29% unstable loci are classified as “microsatellite low” (MSI-L), and “microsatellite stable” (MSS) tumors are marked with no unstable markers [16]. In a study conducted by Mori et al. [18], a comprehensive genomic screening of microsatellite coding regions was performed, revealing the presence of mutations in nine loci (TGF-βR2, Bax, MSH3, ActRIIB, SEC63, AIM2, NADH–ubiquinone oxidoreductase, COBLL1, and EBP1) in over 20% of tumors. It has been observed that the presence of MSI-H in CRC is associated with superior anti-tumor immune response, inhibition of tumor cell growth, and an improved prognosis when compared to patients displaying MSI-L or MSS status. MSI status is a conceivable predictor for the treatment decision strategy method in MSI-H and MSI-L tumors. Furthermore, MSI tumors are more frequently located in the proximal colon, and present as more poorly differentiated cancers [19]. MSI is rarely found in polyps, except in Lynch syndrome, due to germline mutations in one of the MMR genes. MSI is the hallmark of HNPCC or Lynch syndrome and occurs in >95% of HNPCC cases [16].
(iii) CIN nominated as the suppressor pathway phenotype is observed in 70–85% of CRC tumors and is often considered equal to MSS status [20]. CIN is induced by the occasional gain or loss of the whole chromosome during mitosis. Accordingly, CIN tumors are often aneuploid with structural or numerical aberrations. Furthermore, numerous significant events contribute to the development of the CIN, such as encompassing mutations in some oncogenes, such as *APC*, *KRAS*, *TP53*, *CTNNB1*, and *PIK3CA*, and loss of heterozygosity (LOH) chromosome in 18q with some of the tumor suppressor genes, such as *SMAD2*, *SMAD4*, and *DCC*, in the location. Traditionally, CRC develops through the adenoma to carcinoma pathway. *APC* gene mutation and inactivation are early events that are followed by the activation of *KRAS* as an oncogene due to mutation appearance in the adenomatous stage, followed by the inactivation of *TP53* on chromosome 17p and the deletion of chromosome 18q, leading to metastatic carcinoma [21]. *APC* mutations are present at the preliminary stages of neoplasia and are majorly linked with the classic tubular adenoma pathway and CIN cancers [22].
(iv) CIMP is an epigenetic event that has been observed to precede the onset of cancer. The process involves an increase in methylation levels within the promoter region, which can lead to the silencing of tumor-suppressor genes. Conversely, global hypomethylation has been linked to genomic instability and chromosomal abnormalities. In CRC, epigenetic instability manifests as hypermethylation of CpG islands, often in tandem with global DNA hypomethylation. In CIMP-positive CRC, promoter regions of tumor suppressor genes are frequently hypermethylated, resulting in loss-of-function in these genes.

CIMP was described by Toyota et al. [23] as a tumorigenesis pathway in 1999. Furthermore, CIMP tumors coincide to a considerable extent with MSI. CIMP is characterized by high promoter methylation of various genes, such as *p16*, *MINT* clones, *THBS*, and *MLH1*, and is correlated with some significant clinical, pathological, and molecular features, such as female sex, old age, right-sided tumors, high MSI, and *BRAF* V600E mutations [24,25]. CIMP is a subclassification that is determined by the integration of genetic and epigenetic instability, and it is further divided into two categories: CIMP-low and CIMP-high. Analysis of DNA methylation profiles has demonstrated that roughly 20% of CRCs fall into the CIMP category [16].

### 2.3. Supervised Gene Expression Profiles for Prognosis Prediction of Early Stages CRC

Gene expression profiles (GEP) aim to predict the likelihood of tumor recurrence after surgery in the early stages of CRC and could also provide predictive information about the benefit of pharmacological treatment [26]. The commercially available gene expression profile assays include the following (Table 1):

- The Oncotype DX colon cancer assay (Genomic Health, Redwood City, CA, USA, http://www.genomichealth.com (accessed on 27 February 2023) predicts recurrence in stage II colon cancer patients after surgical resection [27,28]. Oncotype DX includes a 12-gene expression assay (7 cancer-related—*BGN*, *C-MYC*, *FAP*, *GADD45B*, *INHBA*, *Ki-67*, *MYBL2*—and 5 reference genes—*ATP5E*, *GPX1*, *PGK1*, *UBB*, *VDAC2* [29]—based on reverse transcriptase-polymerase chain reaction (RT-PCR). It has been tested on archival formalin-fixed and paraffin-embedded (FFPE) tumor tissue specimens in the QUASAR trial [14].
- Coloprint (Agendia, Amsterdam, The Netherlands, http://www.agendia.com (accessed on 27 February 2023) is a test based on an 18-mRNA signature that predicts CRC relapse in the early stages of the disease. Furthermore, Coloprint uses whole-genome expression data analysis and has been validated in several independent cohorts. In addition, ColoPrint can predict the development of distant metastasis in stage II CRC patients and facilitates treatment strategy decisions for patients who may be safely managed without chemotherapy [30].
- A further prognostic gene expression microarray-based assay, ColDx, commercially administered as GeneFx Colon, was developed by Almac (Almac Group Ltd., Craigavon, UK) [31]. It is based on a 634-gene signature panel [32] and performed on FFPE tumor samples [33]. This assay differentiates stage II tumors into low- and high-risk for disease recurrence [14].
- CologuideEX (Oslo University Hospital, Oslo, Norway) stratifies CRC patients into high- and low-recurrence risk groups based on molecular markers. It contains a small panel of 13 genes (*PIGR*, *CXCL13*, *MMP3*, *TUBA1B*, *SESN1*, *AZGP1*, *KLK6*, *EPHA7*, *SEMA3A*, *DSC3*, *CXCL10*, *ENPP3*, and *BNIP3*) optimally combined for robust risk stratification of CRC stage II patients using a consecutive Norwegian test series [34] by Affymetrix exon-based microarrays (Affymetrix, Santa Clara, CA, USA). The test was performed on fresh-frozen samples [35].
- The OncoDefender-CRC test investigates the expression profiles of 5 genes (*BMI1*, *ETV6*, *H3F3B*, *RPS10*, and *VEGFA*) on archival FFPE tumor tissue by RT-PCR using the custom 384-well TaqMan low-density arrays. This dichotomous 5-gene prognostic signature is able to differentiate between patients with lymph node-negative, invasive CRCs at “high risk” from those at “low risk” of recurrence within 3 years after curative resection [36].
- The ColoGuidePro was also created at Oslo University Hospital (Norway) to predict good or poor prognosis in stages II and III of CRC patients. This assay includes a panel of seven genes, and it is based on Affymetrix exon-based microarrays to determine the expression signature of fresh-frozen tissue samples [37].

Despite the availability of these platforms, their use in the clinic is not currently recommended by international guidelines due to unclear clinical utility for risk stratification and a lack of strong validation in predicting treatment benefits. In contrast, there are contradictions among gene expression-based CRC classifications that need to be resolved to correlate cancer cell phenotypic features with clinical behavior and guide targeted treatments.

**Table 1:** Available gene expression assays predictive of prognosis in early-stage CRC.

Assay	Company	Gene Signature	Specimen Used	Technique for GEP	Actual Status	Refs.
ColoPrint colon cancer recurrence assay	Agendia, Inc.	18-gene expression profile	Fresh tissue or fresh, frozen tissue	Agendia customized whole-genome high-density microarrays	Not FDA approved	[38,39,40]
GeneFx Colon	Licensed by Precision Therapeutics (originally developed by Almac Diagnostics as the ColDX assay)	634 probe-set signatures	Formalin-fixed paraffin-embedded tissue	Amplified product hybridized to Almac Colorectal Cancer DSA on Affymetrix 7G scanner	Not FDA approved	[32]
OncoDefender-CRC (colon and rectal cancer)	Everist Genomics	5 genes	Formalin-fixed paraffin-embedded tissue	TaqMan Array	Not FDA approved	[36]
Oncotype DX colon cancer assay	Genomic Health, Inc.	12 genes (7 prognostics and 5 reference genes)	Formalin-fixed paraffin-embedded tissue	Reverse transcription-quantitative PCR (RT-qPCR)	Not FDA approved	[27,28,29]
ColoGuideEx	Oslo University Hospital, Norway	13 genes	Fresh, frozen biopsies	Exon-based microarray	Unknown status	[34]
ColoGuidePro (Derived from ColoGuideEx)	Oslo University Hospital, Norway	7 genes	Fresh, frozen biopsies	Exon-based microarray	Unknown status	[37]

### 2.4. The Consensus Molecular Subtyping as a Transcriptome-Based Staging

In recent years, several unsupervised gene expression-based classifications have been obtained due to the advancements in sequencing methods. Despite the availability of such classifications, their clinical utility has still not been recognized due to technical problems (e.g., the lack of standard methodology, different data processing, and diverse gene expression values, and practical reasons). In 2015, Guinney et al. integrated previously available classifications to obtain a distinct classification of CRC, and, thus, tried to eliminate the discrepancies between previous subtyping systems [41]. The CRC subtyping consortium (CRCSC) normalized and integrated data from 6 previously available CRC subtyping classifications for a total of approximately 4000 primary tumors from 18 CRC datasets, by obtaining 4 distinct subtypes of CRC (CMS1–4) characterized by different prognoses, i.e., the “consensus molecular subtype (CMS)” [41] (Table 2). 

CRCSC was established to assess the core subtypes’ fundamental molecular features of CRC among the previously available gene expression-based classifications and to merge all the accessible data sources, such as mutations, methylation status, copy-number variations, microRNAs, and proteomics, to examine whether CMS can be extensively utilized in clinical practice approach [41]. Then, they determined the biological and molecular features of each subtype and ultimately assessed the prognostic and clinical association of the CMS subtypes. The CMS stratification named as a “gold standard” consists of CMS1 (immune subtype, 14%), CMS2 (canonical subtype, 37%), CMS3 (metabolic subtype, 13%), and CMS4 (mesenchymal subtype, 23%). Heterogeneous samples with mixed characteristics are classified as mixed or indeterminate samples (14%) [41].

In terms of biological features, considering the genomic aberrations, CMS1 comprised the more significant number of MSI tumors that demonstrated the hypermethylation status. They were also hyper-mutated samples with a low prevalence of somatic copy-number alterations. Likewise, they had over-expression of proteins involved in DNA damage repair. CMS1 is the immune subtype with high expression levels of genes involved in the immune response. In CMS1, an enhanced expression of genes involved in the diffuse immune infiltration, comprising TH1 and cytotoxic T cells, accompanied the immune invasion pathways. This subtype is widely hypermethylated and has a low prevalence of SCNAs [42].

CMS2 or “canonical subtype” was characterized by epithelial differentiation and has the highest distribution of tumors with high copy number counts in oncogenes and low copy number counts in tumor suppressor genes. Marked activation of WNT and MYC signaling, typical of CRC carcinogenesis was also present [41].

CMS3 tumors were defined as CIN tumors with fewer SCNAs and higher CIMP status. In addition, almost 30% of CMS3 tumors were hyper-mutated that overlapped with other MSI phenotypes [41]. The CMS3 or “metabolic subtype” indicates epithelial features and metabolic deregulation. 

Ultimately, CMS4, or the “mesenchymal subtype”, comprises mesenchymal-like tumors with high stromal infiltration in combination with normal cells [43]. A significant up-regulation of genes involved in epithelial–mesenchymal transition (EMT) and of signatures related to the TGβ signaling activation, angiogenesis, matrix remodeling pathways, and the complement inflammatory system has been observed in CMS4 [41].

CMS4 relates to poorer patient prognosis and poor response to anti-EGFR drugs and routine chemotherapy regimens. Additionally, CMS4 is correlated with worse disease-free and overall survival, with the highest tendency to develop distant metastasis among other subtypes [44].

Overall, CMS2–4 have revealed an increased level of CIN [41].

The integrative analysis of mutations and copy-number variations based on The Cancer Genome Atlas (TCGA) data showed that *BRAF* mutations are frequently found in CMS1 and related to the MSI phenotype. *KRAS* mutations are frequently seen in CMS3. Additionally, the receptor tyrosine kinase (RTK) and mitogen-activated protein kinase (MAPK) pathways are generally activated in CMS1 and CMS3. However, none of these genetic aberrations exclusively belong to specific CMS subtypes. The observed heterogeneity in CMS status among CRCs with commonly accepted driver events underscores the significant variability in the biological behavior of such tumors. Additionally, this highlights the poor correlation between genotype and phenotype in CRC [41].

In a molecular analysis of the AGITG MAX clinical trial, there was no significant difference in the proportion of *RAS* mutations across CMS groups, but the incidence of *BRAF* V600E mutation was diagnosed with the highest proportion in CMS1 (34%) compared to CMS2 and CMS4 (each <2%). MSI was not common in this CMS sub-study and was found to be 7% in both CMS1 and CMS3. Finally, CMS1 included a high percentage (39%) of CIMP-high tumors in comparison with CMS2 (3%) and CMS4 (6%) [45].

In association with the clinical variables, several findings have been revealed. The CMS1 group is generally right-sided, presents mainly in women with a greater histopathological grade, and patients in this subtype showed poor survival after recurrence. In contrast, CMS2 subtypes are mostly left-sided, with a higher survival rate after relapse. CMS4 tumors are mainly related to AJCC stages III and IV, and patients with CMS4 CRC display poorer overall and relapse-free survival. Consequently, the poor prognosis after relapse is relevant for patients with MSI and *BRAF* mutations [41]. Therefore, it has been suggested that during the time of transition from the adenoma stage to the advanced carcinoma phase, cells change from CMS1–3 to CMS4 [46]. Most peritoneal metastases have been observed in CMS4 due to colorectal peritoneal carcinomatosis and adverse histopathological features. These observations comprised a high stroma level in both primary and metastatic tumors, inadequate differentiation grade, and increased tumor budding in primary tumors [47].

Individuals with inflammatory bowel diseases (IBD) are at an increased risk for CRC development. The signaling pathways involved in colitis-associated cancer are believed to be similar to those observed in sporadic CRCs but with a distinct sequence of events. Recently, the molecular signature of these cancers has been identified in a study that shed light on the lack of CMS2 tumors among IBD-CRCs, which were, instead, skewed toward CMS4 [48].

Generally, it is believed that the CMSs of CRC may better inform clinicians about prognosis, treatment response, and potential novel therapeutic strategies [41].

## 3. CMS Classification and Regulatory Networks

Currently, the subtyping of CRC using gene expression data does not consider regulatory networks, which makes it difficult to identify the underlying determinants [49].

Molecular characteristics, such as mutation status or MSI, are insufficient to differentiate between the different CRC subtypes, indicating that the driving forces for specific cancer subtypes are more complex. The predominant regulatory networks that contribute to differences in gene expression between the various CRC subtypes are transcription factors (TFs), methylation marks, and microRNAs (miRs). 

Since its publication, the CMS classification has been exploited in different CRC settings (i.e., case series and in vivo and/or in vitro models). An example is represented by the study of Fessler et al. [50], in which through a complex network analysis including miRNAs as putative subtype-specific regulators, but not limited to, differences between CMS1–3 and CMS4 were investigated. The analysis of both mRNAs and miRNAs within the same population is a highly effective approach to elucidate the target genes of miRs and to identify perturbed biological pathways and regulatory networks. This is due to the fact that a single miR has the capacity to regulate multiple mRNAs. Interestingly, the miRNA network that was significantly more expressed in CMS1–3 compared with CMS4 was the most powerful determinant of gene expression regulation according to subtypes [50]. MicroRNA analysis identified the up-regulation of the miR-17–92 cluster that is a direct target of *MYC* in CMS2 tumors [51], and a low expression of the let-7 miR family associated with high KRAS expression levels in CMS3 [41]. In the CMS4 subtype, the miR-200 family that is associated with the EMT regulation was found to be down-regulated. The released data showed that miR-200 promoter methylation is an independent prognostic factor in the AMC-AJCCII-90 dataset [50]. The methylation status of the two promoter regions of miR-200 has been found to have significance in specifying tumors belonging to the mesenchymal subtype, as well as in predicting disease-free survival in stage II CRC patients. This knowledge may be applied in the selection of individuals who could benefit from more aggressive therapy. Notably, patients with highly methylated tumors have been observed to have significantly worse disease-free survival rates in comparison to those with lower methylation levels of the miR-200 loci. Fessler et al. [50] accordingly demonstrated that the epigenetic switching off of the miR-200 family is observed in epithelial CRC cell lines belonging to mesenchymal CMS [50]. Furthermore, the miR-200 family, which inhibits the EMT process, was found to be one of the most abundant and significantly up-regulated miRNAs in the colon-like samples. The patients with CMS4 displayed gradient DNA methylation for both loci, in which samples were enriched in the quartile displaying the highest DNA methylation. Furthermore, the combined methylation of the miR-200 loci was able to predict CMS4 affiliation with high confidence [50]. However, no association was reported between specific miRs and the other three tumor subtypes. A recent study described microRNA profiling according to CRC CMS subtypes as well as mRNA profiling. Three microRNA subtypes were described: miR-LS was correlated with the low-stroma/CMS2-subtype, miR-MI with the mucinous-MSI/CMS1-subtype, and miR-HS with the high-stroma/CMS4-subtype. MicroRNA novel subtypes and their relationships with the CMS classification were validated by use of TGCA data. Hence, miR profiles classify CRC by transcriptional profiling, and, thus, through a real-time analysis that facilitates the prediction of the disease course and treatment response [52].

Therefore, regulatory networks are considered a robust strategy to specify drivers of distinct CRC subtypes, which possess the ability to recognize subtype affiliation and to shed light on the biological behavior of the tumor.

## 4. CMS and Polyps

The increased incidence of CRC stimulated interest and research aimed at providing insights into the molecular diversity of colorectal polyps and precursor lesions to improve personalized and targeted prevention approaches [53]. Molecular understanding of familial and sporadic CRC has been critical in advancing clinical approaches in both early and advanced stages of CRC. Additionally, it is fundamental to understand the molecular diversity of colorectal polyps to better reach the targeted strategies for CRC prevention [54].

Indeed, the lack of knowledge of epigenetic and transcriptomic modifications in benign polyps or precursor adenomas makes CRC limited to targeting prevention strategies.

Inherited syndromes, including “familial adenomatous polyposis” (FAP) and Lynch syndrome, comprise approximately 5% of all CRC cases. FAP-associated adenomas arise from *APC* germline mutation that can switch on WNT/beta-catenin mediated transcription, stimulating the transition of intestinal crypts to precursor lesions, such as tubular or villous adenomas. Moreover, the molecular characteristics of FAP syndrome are similar to CRC with CIN status [55]. Lynch syndrome-related CRC is related to germline mutations in MMR genes (*MLH1*, *MSH2*, *MSH6*, *PMS2*), which, furthermore, evolve in more advanced premalignant lesions. The MSI phenotype prevails in merely half of Lynch syndrome polyps. One-third of all CRC cases are caused by serrated pathways encompassing sessile serrated adenomas (SSA) and hyperplastic polyps (HP). SSAs are mostly right-sided with high CpG island methylation status. In addition, *BRAF* V600E mutation is the prominent mechanism either in sporadic or hereditary CRC [54]. Chang et al. [54] applied CMS stratification to original cohorts of sporadic and familial CRC and 11 GEO datasets. They conducted gene-set enrichment analysis (GSEA) by employing previous biological pathways and expression signatures related to CRC carcinogenesis and observed that CMS1-related polyps were distinguished by considerable enrichment of genes affected in immune and stromal infiltration, in addition to the immune cytotoxicity pathways. Moreover, they indicated robust activation of JAK-STAT and MAPK signaling. CMS2 polyps exhibited significant enrichment for WNT and MYC pathways, which are classical carcinogenesis pathways in CRC. In addition, a small number of polyps were categorized as CMS3. Finally, despite the small number of CMS4-like polyps, they displayed a significant number of mesenchymal and stromal signatures associated with TGFβ activation. Overall, Chang et al. [54] clarified that genes involved in the immune activation and classical carcinogenic pathways were mostly represented in CRC premalignancy. These authors [54], therefore, evaluated the CMS proportion through numerous clinical and molecular features. They found that there was the same distribution of CMS1 and CMS2 among both sexes, and there was no association between the high grade dysplasia/carcinoma in situ and the CMS stratification. In both familial and sporadic CRC, CMS1-like polyps were mainly right-sided. Contrarily, CMS2 polyps were regularly observed in the left colon. Subsequently, they proposed that CMS1 may mainly arise from HP and SSA. In addition, *BRAF* V600E was primarily observed in CMS1 polyps. This analysis failed to show a meaningful representation of *KRAS* in CMS3 due to the small quantity of CMS3 polyps [54]. Chang and his colleagues also found an epithelial canonical CMS2 status in addition to significant WNT and MYC downstream targets enrichment. MMR deficiency was detected at diagnosis in half of the Lynch Syndrome polyps, whereas it was observed in all the advanced adenomas and carcinomas. This study proposes a hypothesis regarding the molecular characteristics of adenomatous polyps from sporadic or hereditary cases, including Lynch syndrome, as compared to hyperplastic and serrated polyps. Specifically, the hypothesis suggests that adenomatous polyps exhibit a CMS2-like phenotype with activation of the WNT and MYC pathways, while hyperplastic and serrated polyps display a CMS1-like phenotype characterized by prominent immune activation. Based on these findings, the study presents a model of pathway activation associated with CMS classification in the development of CRC. The model suggests that while adenomatous polyps are largely classified as CMS2, most hyperplastic and serrated polyps fall under the CMS1 category and may transit into other CMS groups during their evolution into carcinomas.

**Figure 1:**
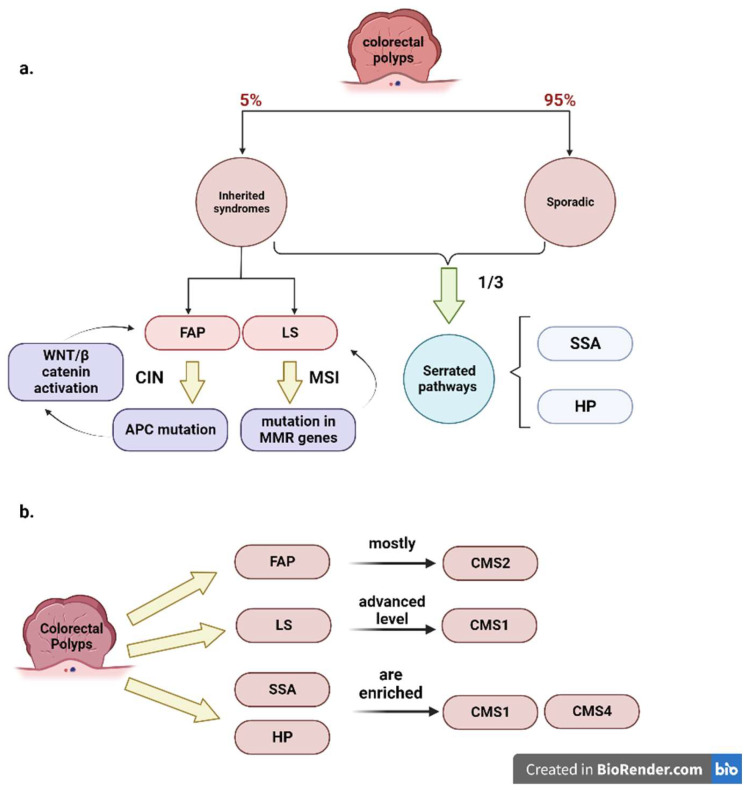
Different types of colorectal polyps and their association with the CMS of CRC. (**a**) Colorectal polyps have a hereditary and sporadic basis. Only 5% of colorectal polyps are familial, consisting of familial adenomatous polyposis (FAP) and Lynch syndrome (LS). FAP-associated adenomas originate from APC germline mutation and are followed by the WNT/B catenin activation, and they are like the CRC with CIN status. Conversely, LS-related adenomas result from a germline mutation in MMR genes and are likely to cause a more advanced premalignancy. The MSI phenotype can be seen in only half of the LS polyps. About one-third of all CRC cases follow the serrated pathways consisting of sessile serrated adenomas (SSA) and hyperplastic polyps (HP). (**b**) Most of the FAP polyps are classified into CMS2. The LS adenomas at the advanced level are transformed from the CMS2 to the CMS1. SSA and HP adenomas are enriched with CMS1 and CMS4 phenotypes. Created with BioRender.com.

Collectively, the study of Chang et al. revealed that most of the FAP-related and sporadic adenomatous polyps are characterized by a CMS2 status, while Lynch syndrome-like polyps are characterized by the CMS1 (MSI-Immune) phenotype. Ultimately, SSA and HP are enriched for either CMS1 or CMS4 (mesenchymal-like) phenotypes [54] (Figure 1).

Fessler et al. [56] utilized organoid cultures from SSA tissues and tubular adenoma (TA) polyps, and obtained an organoid culture carrying the *BRAF* V600E mutation by using the CRISPR/CAS9 procedure. They demonstrated that TGFβ plays a crucial role in the progression of the SSAs to the CMS4 associated with a poor prognosis. Furthermore, they indicated that in tubular adenomas, death by apoptosis is the dominant response to TGFβ. Conversely, induction of a mesenchymal phenotype upon TGFβ treatment prevails in the genetically engineered organoid culture carrying a *BRAF* V600E mutation, establishing a model strategy for SSAs. Finally, Fessler et al. [56] showed that TGFβ signaling is already active in SSA precursor lesions, and that TGFβ is fundamental for leading SSAs to the mesenchymal CMS4 subtype.

Komor et al. [57] also examined the possibility of classifying colorectal adenomas into CMS. They stratified adenomas from TCGA into CMS subtypes. A total of 87% of adenomas were classified into the CMS subtypes. There was a greater prevalence of CMS3 in adenomas than in CRCs (73% vs. 13%).

The CMS3 “metabolic subtype”, which is the least common among CRCs, was the most prevalent among adenomas (*n* = 45; 73%). Only 2% of the adenomas were stratified into the CMS1 subtype, and 13% were classified as the CMS2 subtype. There was no adenoma belonging to the CMS4 subtype due to the lack of stromal invasion in adenomas. CMS3 was associated with the adenomas at low risk of developing CRC, while high-risk adenomas were investigated in CMS2 and CMS1 [57].

Overall, a transition of CMS groups occurs in colorectal premalignant lesions and may help guide the development of future biomarkers or preventive treatments for CRC.

## 5. Role of CMS in the Prediction of CRC Prognosis

To translate CMS stratification into clinical approaches, various studies have been carried out in recent years. The main objective of these studies was to apply CMS to independent cohorts to validate its prognostic role. Lenz et al. [58] evaluated CMS and other somatic biomarkers in patients from the LUME-Colon 1 study, a placebo-controlled phase III trial of nintedanib in advanced CRC. The correlation of CMS, tumor genomic, and circulating biomarkers with the clinical outcomes were assessed in 231 out of 245 patient samples in the tumor tissue dataset, as 231 (94.3%) had evaluable RNA-Seq gene expression data and met the necessary quality criteria for CMS classification in advanced CRC cases. The distribution of patients among the CMS subtypes was as follows: CMS1 (1.7%), CMS2 (27.7%), CMS3 (0.9%), and CMS4 (51.5%), whereas 18% were referred to as unclassified or mixed CMS. The meaningful difference between the CMS percentages in this study and previously reported data may be due to certain factors, such as tumor locations, stages of the disease, and previous drug treatment lines.

Overall, compared with CMS2 or CMS4, unclassified/mixed CMS was associated with prolonged overall survival in the nintedanib arm. Furthermore, gene expression-based pathway analysis for nintedanib indicated a relation between vascular endothelial growth factor (VEGF)-related signaling and overall survival. These results propose a potential for adequate nintedanib treatment response in unclassified/mixed CMS [58].

The integration of circulating tumor DNA (ctDNA) and CMS classification into the postoperative management of stage III colon cancer has been explored in two prospective studies. In the study by Li et al. [59], it was determined that the combined use of postoperative ctDNA and CMS classification yielded a more accurate prediction of outcomes for stage III colon cancers. The study of Tarazona et al. [60] failed, instead, to identify a relationship between postoperative plasma ctDNA, CMS, and prognosis, since only the detection of postoperative plasma ctDNA and the absence of *CDX2* expression were identified as prognostic indicators for recurrence in patients with localized colon cancer. Both these findings need to be validated. Afterwards, it will be possible to establish if the integration of ctDNA and CMS classification may help in the prediction of CRC prognosis.

Interestingly, Becht et al. [61] retrospectively quantified immune and stromal infiltration of CMS1–4 subtypes in CRC patients. A strong correlation between the tumor cell phenotypic behavior and the composition and functional orientation of the immune microenvironment was reported. Specifically, the mesenchymal tumors’ association with a proinflammatory, proangiogenic, and immunosuppressive microenvironment is reported dynamically. Furthermore, results showed that two subgroups were designated by the high expression of immune signatures: the expected MSI-rich CMS1 group and the unexpected mesenchymal CMS4 group. This classification stratifies CRC into intrinsic subtypes with different prognoses. CMS2 and CMS3 were supposed to be immunogenetically “cold” due to the absence of T cells infiltrating the tumor [62] and the decreased expression of the major histocompatibility complex (MHC1) gene. Conversely, the overexpression of genes involved in cytotoxic lymphocytes present in CMS1 is associated with a good prognosis. Likewise, CMS4 showed a high expression level of lymphocytes, monocytes, and signature factors of angiogenesis, inflammation, and immunosuppression, and this has been associated with a poorer prognosis [61].

In addition, Smeby et al. [63] analyzed CRC stage I–IV for MSI and mutation status in *KRAS* hotspots (codons 12, 13, and 61) and *BRAF* (codon 600). The poorest prognostic impact of *BRAF* V600E mutation in MSS tumors was found in CMS1 patients, and the overall survival rate was significantly lower than that in wild-type tumors, irrespective of tumor location. Since then, enrichment of metastatic disease in *BRAF* V600E-mutated CMS1 MSS tumors was correlated to the early and late stages of disease. MSI-H and MSI-L tumors did not show prognostic association with *BRAF* mutation within any of the CMS subtypes. Consequently, the prognostic impact of *BRAF* V600E mutations was considered to be highly dependent on MSI status within CMS1 [63].

Available information suggests epigenetic age acceleration (i.e., the difference between DNA methylation age and chronologic age, the “epigenetic clock”) as a new biomarker for evaluating cancer risks and disease prognosis [64]. In 2019, a study by Zheng et al. [64] retrospectively assessed the correlation between epigenetic age acceleration and CRC CMS subtypes. Using Horvath’s age prediction model, they assessed the epigenetic clock of 345 CRC cases from TCGA and then associated the obtained results with CMS classification and clinical outcome (i.e., overall survival). Although the epigenetic age was not significantly associated with survival after univariate analysis, shorter survival was associated with epigenetic age in the CMS4 subtype. Overall, no significant association was observed with mortality in a univariate analysis of epigenetic age acceleration, but age and tumor stage were distinguished as significant predictors of survival [64]. Furthermore, the analysis of age, tumor stage, and molecular subtype revealed no significant correlation for epigenetic age acceleration, except for patients detected in the stage II CRC and CMS4 subtype. In addition, uni- and multi-variate analyses adjusted for age and tumor stage illustrated worse survival with an increment in epigenetic age acceleration in CMS4 CRC [64].

Purcell et al. [8] investigated the role of CMS as a prognostic tool for CRC and compared standard histological classification with CMS. They assessed molecular subtyping related to clinical/pathological aspects and patient outcomes in a large single-institution cohort of treatment-naïve CRC tumors and compared the results to the standard histological classification. CMS subtypes were distributed in CMS1 (19%), CMS2 (47%), CMS3 (12%), and CMS4 (6%). By designating both TNM staging and CMS in survival analysis models, the authors concluded that only TNM staging independently of age and gender was significantly related to mortality, whereas CMS could not. Furthermore, progression-free survival and overall survival were significantly related to the CMS subtype after age and sex adjustment, and again no significant association between CMS stage and overall survival was observed [8]. Therefore, the finding of this study [8] revealed that as a prognostic indicator, CMS stratification does not outperform TNM staging, but gene expression-based subtyping could improve prognostication in stage II CRC.

## 6. In Vitro and In Vivo CMS-Based Studies for Tailoring Treatments in CRC

The ability to predict treatment response is becoming increasingly necessary to obtain the maximum clinical benefit and to reduce healthcare costs. Hence, cell lines open a horizon for investigating new drugs and predictive biomarkers. Moreover, patient-derived xenografts (PDXs), produced by transplanting primary tumor fragments into mice, represent a valid experimental option [65]. CMS stratification has demonstrated an association between CRC and clinical features in both in vitro and in vivo.

**Figure 2:**
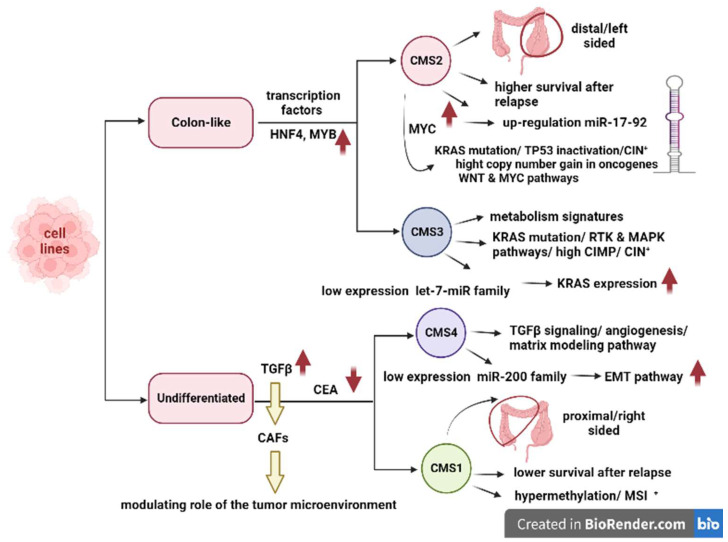
Colon cells in relation to the CMS subtypes and some molecular and genomics features of each subtype. “Colon-like” and “undifferentiated”. Colon-like cell lines include both CMS2 and CMS3, representing an increased number of key transcription factors (HNF4A and MYB). Undifferentiated cell lines include all CMS4, and most CMS1, which have an elevated expression of TGFβ-provoked genes. This TGFβ signaling in cancer-associated fibroblasts (CAFs) induces the initiating capacity for CRC cells. Furthermore, the occurrence of premetastatic features of the CAFs through TGFβ 1/2 paracrine signaling indicates the modulating role of the tumor microenvironment in cancer cell expression. Created with BioRender.com.

Two specific subgroups of 34 CRC cell lines labeled “colon-like” (including, for instance, HT29/WiDR, SW1116, SW403, SW948, and CL-34) and “undifferentiated” (including, for instance, CaCo2, DLD-1/HCT15, SW48, SW480/SW620, and HCT116) were noticeable at the mRNA, miRNA, and protein levels [66]. Colon-like cell lines encompassed both CMS2 and CMS3 and expressed an increased number of gastrointestinal marker genes, such as key transcription factors (HNF4A and MYB). HNF4A is considered a stimulant for the 20q13.12 focal amplification peak, proposing a correlation between overexpression and expression subtype (Figure 2) [66]. Moreover, colon-like cell lines had a unique expression of mir-194 and mir-192 as a distinctive marker between these two cell lines [67].

Undifferentiated cell lines comprised all CMS4 and most CMS1, indicating a more stromal signature [66]. Furthermore, undifferentiated cells had a high expression of TGFβ-provoked genes containing TGFβ1/2 cytokines. It has been recognized that CRC tumors with increased expression of TGFβ have a great potential for metastasis. In addition, TGFβ signaling in cancer-associated fibroblasts (CAFs) stimulates the initiating capacity for CRC cells [68]. Premetastatic features of the CAFs occur through TGFβ1/2 paracrine signaling, indicating the modulating role of the tumor microenvironment in cancer cell expression. Likewise, in undifferentiated cell lines, carcinoembryonic antigen (CEA) expression is lower than in colon-like cell lines. These data suggest that the CEA biomarker is not as advantageous for CMS1 and CMS4 patients [66].

Patient-derived organoids (PDOs) are 3D cell culture models that can be derived from patient tumors to capture the genetic and phenotypic heterogeneity of the original tumor. The use of PDOs in combination with CRC CMS classification has been explored in several studies for predicting drug response. For instance, Mo et al. [69] generated PDOs from primary tumors and paired liver metastases of mCRC patients and demonstrated their utility in predicting the response to chemotherapy (FOLFOX or FOLFIRI) in these patients [69]. Similarly, Laoukili et al. [70] linked oxaliplatin resistance of peritoneal metastases in CRC to CMS4 status and high reducing capacity. Michels et al. [71] investigated human colon organoids and found that oncogenic Wnt signals are associated with good prognosis in CMS2 tumors, whereas receptor-mediated signaling is linked to CMS4 tumors and poor prognosis. Fessler et al. [56] used human organoid cultures to show that TGFβ is a critical cue for directing SSAs to the mesenchymal, poor-prognosis CMS4 of CRC. Interestingly, Mazzeschi et al. [72] showed in PDOs, as well as in an in vivo xenograft mouse model, that CMS classification may help to predict the efficacy of ALK targeting, with CMS1 being the more responsive subtype to ALK inhibitors. In summary, combining CMS classification with PDOs holds promise for improving the prediction of drug response in CRC patients. However, future studies are needed to validate these findings in larger patient cohorts and to integrate them into clinical practice for personalized treatment.

The utilization of PDX models involves the transplantation of small portions of tumor tissue from surgical patients into immunodeficient mice [73]. Several investigations have highlighted the potential of PDX models as a preclinical tool for drug screening due to their ability to accurately predict and recapitulate patient drug responses in CRC [74]. In 2017, Isella et al. [75] pointed to the impact of stromal content on the transcriptional classification of CRC. As is known, the PDX stromal cells of human cancer are substituted by murine counterparts as a consequence of xenotransplantation. Isella et al. [75] deployed human-specific expression profiling of CRC PDXs to assess cancer cell-intrinsic transcriptional features. Through this approach, five CRC intrinsic subtypes (CRIS) were identified. They were characterized by distinctive molecular, functional, and phenotypic peculiarities. CRIS subtypes successfully categorized independent sets of primary and metastatic CRCs, with limited overlap on existing transcriptional classes and relevant predictive and prognostic performances [75]. The observed drug responses in the xenograft model were consistent with those in tissue slice cultures performed in vitro.

Hence, there is significant overlap between CRIS and CMS subtypes, although there are some differences as well. As a matter of fact, CMS and CRIS classification strategies combine differential biological activity between tumors beyond single gene mutations, and can also be linked to drug sensitivity and resistance for specific subtypes, thus, providing an attractive method of stratifying colon cancer patients [76]. An interesting study by Linnekamp et al. [65] aimed at determining the heterogeneity and evaluating CMSs in CRC cell lines, primary cultures, and PDX. They established a repository and classified their models according to the four CMSs, independent of the stromal contribution. The validation of CMS classification was performed by functional analysis. In this context, the mesenchymal abundance in CMS4 and metabolic dysregulation in CMS3 were demonstrated. An apparent distinction in sensitivity to chemotherapy-induced apoptosis, specifically between CMS2 and CMS4, was identified [65]. CMS2 xenograft growth was delayed by chemotherapy, whereas this did not occur in CMS4 xenografts. Conclusively, these data demonstrated that molecular subtypes in CRC cell cultures and PDXs display tumor cell-intrinsic and stable features. CMS4 was permanently discovered in all models, which can be characterized as a tumor cell-intrinsic phenotype in addition to stromal cell accumulation. This context proved a platform of heterogeneity in CRC [65].

## 7. Role of CMS in the Prediction of Drug Response in CRC

Cancer drug treatment goals are to cure the disease, prolong survival parameters, improve symptoms of disease, and maintain a good quality of life for as long as possible. The median overall survival of metastatic CRC (mCRC) patients suffering from an unresectable disease may reach 30 months when utilizing cytotoxic chemotherapy associated with targeted therapies, such as anti-EGFR antibodies and anti-angiogenic drugs or immunotherapy [77]. Nevertheless, the selection of patients who may highly benefit from a specific treatment regimen is still challenging. Biomarkers able to predict the chemotherapy’s efficacy and spare the patients from excessive toxicity may strongly contribute to this goal [78,79]. Currently, first-line treatment relies on a fluoropyrimidine-based cytotoxic chemotherapy combination in association with a biological and/or targeted agent, according to the individual molecular status [80,81].

The predictive role of CMS in drug treatment response has been evaluated in a relevant number of clinical studies. Overall, in both adjuvant and metastatic settings, data related to a potential benefit from a molecular subtype approach are principally based on retrospective datasets, and prospective confirmation will grant personalized treatment in the future.

Although the prognostic role of CMS in retrospective studies has been confirmed in the metastatic setting, the best prognosis belongs to CMS2, whereas CMS1-designated tumors present a higher risk of disease progression and death after chemotherapy. Similarly, CMS1 responds to immunotherapy according to mesenchymal features and immunosuppressive molecules, whereas CMS4 has a poorer prognosis. Accordingly, patients with a CMS1 signature may benefit from immune checkpoint inhibitors regardless of MSI status. These comprehensive analyses may contribute to the switch from “one marker–one drug” to “multi-marker drug combinations” allowing oncologists to give “the right drug to the right patient” [82]. The revolution in mCRC treatment will be facilitated by the deeper characterization of dynamic integration of multi-omics as genomic and transcriptomic subtypes, encompassing tumor and immune–stromal characteristics to reduce negligible biomarkers and guarantee innovative effective therapeutic strategies [82]. The clinical outcome optimization will be achieved by an integrative and dynamic classification system that connects molecular features to targeted drugs and immunotherapies through the prospect of precision medicine in mCRC [83]. Traditionally, Heidelberger and colleagues [84] described the potential of 5-fluorouracil (5-FU) as the backbone of CRC treatment. Other drugs have been added to 5-FU over the years, starting from 5-FU supplementation with the reduced folate leucovorin, to the 5-FU/leucovorin association with the topoisomerase I inhibitor irinotecan (FOLFIRI) and/or the platinum-coordination complex oxaliplatin (FOLFOX and FOLFOXIRI) respectively. The oral 5-FU prodrug capecitabine may replace 5-FU/leucovorin in these combinations. Biological agents, such as the anti-EGFR monoclonal antibodies cetuximab/panitumumab and the anti-VEGF monoclonal antibodies bevacizumab (BV) and ramucirumab, or the anti-VEGF recombinant fusion protein aflibercept, are added to cytotoxic drugs in mCRC to boost their efficacy [85]. More recently “agnostic” antibodies with histology-independent development models, such as pembrolizumab and nivolumab, i.e., two programmed death receptor-1 (PD-1)-blocking antibodies, as well as larotrectinib and entrectinib, which are indicated in solid tumors displaying the neurotrophic receptor tyrosine kinase (NTRK) gene fusion, have been also approved for the treatment of mCRC.

Okita et al. [78] performed a retrospective study on 193 mCRC patients to elucidate the potential role of CMS as a predictive biomarker of standard treatment. The CMS subtype distribution in this study was as follows: CMS1 (10.9%), CMS2 (27.5%), CMS3 (35.8%), and CMS4 (25.9%). Patients underwent first-line chemotherapy including irinotecan or oxaliplatin with or without anti-EGFR/VEGF monoclonal antibodies. Overall, the irinotecan-treated group showed a better therapeutic response compared with the oxaliplatin-treated group. In relation to CMS classification, patients with CRC classified as CMS4 who received irinotecan-based chemotherapy obtained a better response (objective response and survival parameters) compared with CMS4 tumors treated with oxaliplatin-based chemotherapy. In addition, patients with tumors classified as CMS1 and CMS2, treated with cytotoxic chemotherapy (mainly irinotecan-based chemotherapy) combined with anti-EGFR monoclonal antibodies, showed the worse and the best progression-free and overall survival, respectively, among the four subtypes. Overall, these results are in agreement with previous first-line chemotherapy reports [86,87] and contribute to suggesting CMS as a potential factor predictive of drug efficacy. A subanalysis of the FIRE-3 trial carried out by Stinzing et al. [86] evaluated the efficacy of FOLFIRI plus cetuximab or bevacizumab in 592 *KRAS* exon 2 wild-type mCRC patients. Study findings showed CMS as a prognostic factor independent of the type of drug treatment. CMS was associated with a different outcome in *RAS* wild-type mCRC, although a trend toward more favorable survival parameters in all CMSs was mainly observed when patients were treated with cetuximab compared with bevacizumab. This association was, however, statistically significant only for CMS4. Lenz et al. [87] performed a subanalysis of the CALGB 80405 phase 3 trial including 581 mCRC patients treated with FOLFIRI or FOLFOX plus bevacizumab or cetuximab as a first-line therapy. As for the above-described study, results showed that CMS subtypes were highly prognostic for survival parameters (overall survival and progression-free survival). In this analysis, CMS1 CRC patients treated with bevacizumab had a significantly longer overall survival compared with those treated with cetuximab. The opposite was observed in the CMS2 subtype.

A further study showed that the enterocyte subset (i.e. according to the CRC Assigner classifier, https://github.com/syspremed/CRCAssigner (accessed on 27 February 2023)) of the CMS2 subtype displayed sensitivity against oxaliplatin, whereas all the other subtypes were extensively resistant [88]. Reciprocally, irinotecan shows a potential influence on CMS4-like cancers in metastatic disease [89].

Biomarkers predicting bevacizumab response are not available. Thus, it is not possible to identify patients who are most likely to benefit from it. In a translational post-hoc analysis of the phase III MAX study, Mooi et al. [45] stratified archived primary tumors from 237 patients (50% of AGITG MAX trial population) according to CMS subtypes to evaluate the CMS role as a prognostic and predictive biomarker of bevacizumab benefit in mCRC. MAX was a phase III randomized controlled trial performed by the Australian Gastrointestinal Trials Group (AGITG), which met its primary endpoint of improved progression-free survival by adding bevacizumab to capecitabine-based chemotherapy [90]. In the subsequent analysis, associations of CMS with other molecular and clinical features used to classify CRC, e.g., MSI, *RAS* mutations, *BRAF* mutations, CpG island methylator phenotype (CIMP), and primary tumor location (left versus right), were evaluated.

Collectively, Tebbutt et al. found that CMS2 is associated with the best outcome and CMS1 with the worst. Likewise, a considerable association between CMS and bevacizumab treatment was found, where CMS2 and possibly CMS3 may preferentially benefit from the addition of bevacizumab to capecitabine-based chemotherapy in the first-line treatment of mCRC. These outcomes provide possible insights into the molecular profiles that may condition response to anti-angiogenesis treatments in mCRC and warrant further verification in extra cohorts. Ultimately, among all studies, Mooi et al. [45] proved the possibility of applying the CMS classifier to inform prognosis and treatment selection. Although standard adjuvant treatment (FOLFOX) for stage III is recommended, systemic adjuvant treatment for CMS4 patients does not show any benefit [91]. In metastatic disease, CMS4 patients are resistant to anti-EGFR therapy, regardless of *KRAS* mutation status [92].

In the adjuvant setting (i.e., pMMR high risk stage II and stage III) the standard drug treatment of CRC is represented by the association of a fluoropyrimidine (5-FU or capecitabine, i.e., FOLFOX or CAPOX) and oxaliplatin [93]. Interestingly Song et al. [88] pointed out that only patients with the epithelial CMS2-like subtype and not those with the CMS4-like subtype benefit from adjuvant standard chemotherapy. As is known, only a portion of stage II and III CRC patients respond to adjuvant chemotherapy. Buikhuisen et al. suggested that CMS subtype-specific sensitivities can potentially explain this variation [76]. Alternative chemotherapeutic treatments were suggested for the stem-like colon cancer subtype, which to some extent resembles the CMS4 subtype. Two independent clinical trials showed the superiority of FOLFIRI over FOLFOX in CMS4 [76,89]. Allen et al. [94] reported that CRC patients who were categorized in the CMS2 subtype significantly took advantage of adjuvant chemotherapy treatment in both stage II and III (*p* = 0.02 and *p* < 0.001, respectively). However, concerning the CMS3 subtype, only stage III patients benefitted from drug treatment (*p* = 0.001). Following CRIS classification of the CMS2 subtype, only the CRIS-C subtype significantly benefitted from adjuvant chemotherapy in stage II and III diseases (*p* = 0.0081 and *p* < 0.0001, respectively), while the CRIS-D subtype only benefitted in stage III (*p* = 0.0034) [94].

Overall, these data have been substantially confirmed by a recent meta-analysis that evaluated CMS in relation to adjuvant and metastatic CRC settings [95]. Thus, the CMS classification could contribute, in the future, to the selection of CRC patients and treatments. However, in order to allow for the future implementation of CMS in the clinical setting, or eventually of fewer surrogate CMS biomarkers that may efficiently work as CMS, it will be important to take into consideration some technical aspects that currently have not been standardized. For instance, few prospective trials have been performed, and most of the available CMS data are based on retrospective trials. Furthermore, both fresh-frozen samples and formalin-fixed paraffin-embedded (FFPE) tissues are commonly used, although with a preponderance for FFPE tissues due to their higher availability. Thus, although today, RNA extraction methods from FFPE are more efficient compared with those used in the previous years, this tissue source could affect to some extent the CMS results. Differences in CMS proportion between samples from biopsies and tumor resection have also been evidenced (e.g., a higher percentage of unclassified samples in biopsies) [96]. Intra-patient heterogeneity has been shown to provide different CMS results due to differences among sampled tumor areas [97]. Furthermore, different gene expression platforms are commonly used, and different algorithms may be employed to establish CMS (e.g., the “R CMSclassifier” package and the Pearson correlation-based classifier for population-based studies and single-sample prediction, respectively). Thus, common efforts are needed to achieve the standardization of the CMS classification in order to design powerful translational clinical trials that may be definitely open to the application of CMS in routine clinical practice.

## 8. CMS and Immunoscore

It is widely acknowledged that the immune system may recognize and, thus, eliminate tumor cells in the initial stage of tumor development [98]. The enrichment of tumor-infiltrating lymphocytes has been shown to have significant positive prognostic value regardless of tumor mismatch repair status in local and advanced CRC [99]. The current CRC TNM classification is informative only from the prognostic point of view. Treatment response is, in fact, not predicted. Multiple classifications based on tumor cell characteristics have been suggested. Several scientists have studied the host immune response against cancer and shown the prognostic impact of the tumor-infiltrating immune cells. A new methodology was introduced, namely the “immunoscore”, which has been defined to quantify the in situ immune infiltration [100].

System biology, as well as large-scale analysis, define powerful approaches to identify the mechanisms correlated with tumor progression and tumor recurrence. Integrative analyses uncover the value of immune infiltration in human cancers as the “immune contexture”. The immune contexture is defined as the functional orientation, density, and location of adaptive immune cells within distinct tumor regions [101]. The powerful immune classification derived from immune contexture has been named as the “immunoscore”. The immunoscore term is identified as the numeration of two lymphocyte populations (CD3/CD45RO, CD3/CD8, or CD8/CD45RO), both in the core of the tumor and in the invasive margin of tumors, as a clinically beneficial prognostic marker in CRC. The immunoscore ranges from immunoscore 0 (I0), when low densities of both cell types are found in both regions, to immunoscore 4 (I4), when high densities are found in both regions. The immunoscore is considered a very important prognostic factor for disease-free survival, disease-specific survival, and overall survival, especially at early-stage CRC; on the other hand, it has biological and immunological meaning (adaptive immune response to tumors) and provides an implement for selecting novel therapeutic approaches, such as immunotherapy, i.e., anti-CTLA4, anti-PDCD1 (PD-1) and anti-CD274 (PD-L1) [100].

Although the prognosis of resectable CRC is based on the AJCC and the Union for International Cancer Control (UICC) TNM classification system and tumor cell characteristics, time to recurrence and overall survival are strongly related to the intensity of the immune reaction. In this regard, Franck Pagès et al. [102] pointed to the in situ immune cell infiltration in tumor cells and the association with a favorable prognostic effect. At a glance, they focused on the reproducibility and robustness of the consensus immunoscore as a strong prognostic factor in association with the TNM classification system, and gave specific attention to its implementation as a new component in cancer classification, assigned as TNM-Immune. This would represent the first standardized immune-based assay for the classification of cancer [100,102]. Haasnoot et al. [46] conducted an extensive multicenter cohort survey of patients with non-pedunculated T1 CRC to assess the regular distribution of microsatellite (MS)-status, CMS, and immunoscore. Likewise, they examined the association with currently used histologic markers for T1 CRC. They also examined the potential for these molecular- and immune-based scores to evaluate the clinical value in the identification of high-risk patients, relying on their prediction of a negative outcome, i.e., lymph node metastasis (LNM) or recurrence. They selected all patients with known lymph node status after surgical resection, occurring in a cohort of 651 patients with non-pedunculated T1 CRC. It has been reported that a small number of CD3 and CD8 T cells infiltrating the tumor’s center and invasive margin, namely a low immunoscore, has been linked with a poorer prognosis in CRC cases [102,103]. Nevertheless, these risk stratification scores have been developed and assessed in patients with generally progressed tumor stages (T2–T4) or lymph node or distant metastasis (stage III or IV, respectively), while their prevalence in T1 CRC is unknown [46]. Conventional histologic markers, such as poor differentiation, lymphovascular invasion, deep submucosal invasion, and tumor budding, currently serve as the basis for risk stratification of T1 CRC and guide the decision to perform additional surgery. However, the lack of specificity associated with these markers can lead to unnecessary surgery for patients classified as high-risk but who do not have LNM (false positives). The validation of newer classification systems in T1 CRC patients, specifically CMS and immunoscore, has the potential to enable the conservation of organs in select patients [46].

T helper type 1 (Th1) adaptive immunity is considered an important prognostic factor. Th1 cells have a vital role in initiating and conserving a practical CD8+ cytotoxic T cell response [104], in the employment of CD8+ cells to the tumor bed, and indirectly mediating immunological tumor cell death [105]. Th1 cells recognize antigens in association with primary histocompatibility complex class II (MHC-II) molecules, which secrete the inflammatory cytokine interferon (IFN)-γ, stimulating class II up-regulation on tumor cells. The majority of immunogenic neo-epitopes are class II-prohibited [106]. Tumor cells evade cytotoxic immune responses by expressing the organized death-ligand 1 (PD-L1) that promptly provokes the PD-1 adverse feedback pathway [107]. The PD-1 checkpoint may be hampered by utilizing anti-PD-1 or anti-PD-L1 antibodies that block interactions between the PD-1 receptor and its ligand PD-L1. Nevertheless, the strategy has only been effective in microsatellite unstable (MSI-high) CRC, such as those that have a great neo-antigen burden that can provoke microenvironmental immunological reactivity. Class II expression on cancer cells is undoubtedly vital in the efficacy of checkpoint blockades. Moreover, in vitro PD-L1 blockade enhances Th1-mediated cytotoxicity only against cells that convey high class II molecules. Hence, an effective immune response is critically dependent on a neo-antigen demonstration by MHC-II molecules [108].

Loupakis et al. [109] reported that CMS1 cases were the first subtype by defining the MS-status with antibodies against MLH1, MSH2, MSH6, and PMS2. To further distinguish CMS2/CMS3 from CMS4 patients, a panel of four immunohistochemical stains was utilized with antibodies against CDX2, FRMD6, HTR2B, and ZEB1, in combination with pan-cytokeratin. Tumors were classified into distinct immune subtypes by immunohistochemistry with antibodies against CD3 and CD8 formulated by Galon et al. [100]. Haasnoot et al. [46] also centrally surveyed tumor-slides and established and immunostained tissue microarrays to determine MSI, CMS (MSI/CMS1, CMS2/3, or CMS4), and immunoscore (I-low/I-high). The association of MSI, CMS, and immunoscore with LNM or recurrence was assessed and adapted for conventional histologic risk factors. Tumors of the 223 CRC patients had 7.1% MSI/CMS1, 91.0% CMS2/3, 1.8% CMS4, and 25% I-low. In addition, patients with CMS4 tumors had a high risk for LNM or recurrence compared with tumors of other CMSs. Although not significant, the risk of LNM or recurrence for tumors with MSI was lower than for other tumor subtypes, while the risk for tumors with a low immunoscore was higher [46].

In the study on the use of CMS and CRIS classifications to predict adjuvant treatment response in stage II–III CRC in order to better predict CRIS-C patients (i.e., the subtype that significantly benefitted from adjuvant chemotherapy in stage II–III CRC) at higher risk of relapse after surgery, Allen et al. [94] evaluated T cell infiltration in specific tumor regions as well as CD8 and CD3 levels. Results showed that CRIS-C patients with low levels of CD8+ tumor-infiltrating lymphocytes were most at risk of relapse in stage II–III disease [94]. Lal et al. [108] examined the immunobiological impacts of *KRAS* mutation in the CMS classification based on the TCGA RNA-seq and the KFSYSCC microarray datasets. *KRAS* mutation is a canonical mutation in CRC, observed at varying frequencies in all CMSs. They demonstrated that *KRAS* mutation is related to good suppression of Th1 cell and cytotoxic cell immunity independently of mismatch repair status, tumor location, neoantigen load, and gene expression-based subtype. Nevertheless, it is reported that the accumulative consequence depends on the CMS in which the mutation is detected. Immunity in *KRAS* mutant CMS2 is more suppressed than in CMS1 and CMS4 compared to *KRAS* wild-type CMS2. Their results clarified classification factors for immunotherapy examination entry in CRC and suggested immunotherapeutic methods to examine *KRAS* mutant patients. The Th1-centric coordinate immune response cluster (CIRC), including cytotoxic T cells, neutrophils, and the interferon gamma pathway, was found to be reduced in *KRAS* mutant CRC in two independent datasets. The mRNA and protein expression of STAT1 and CXCL10 were also decreased in *KRAS* mutant CRC. Multivariate analysis revealed that *KRAS* mutation, along with CMS2 and CMS3, were independent predictors of reduced CIRC expression. The immune response in *KRAS* mutant CRC was heterogeneous, with CMS2 *KRAS* mutant samples exhibiting the lowest CIRC expression, reduced interferon gamma pathway expression, STAT1, CXCL10, and decreased infiltration of cytotoxic cells and neutrophils compared to CMS1, CMS4, and CMS2 *KRAS* wild-type samples in the TCGA dataset [108].

## 9. CMS and Immunotherapy

In recent years, new therapeutic approaches and better therapeutic strategy selection have found application in CRC patients and have improved the outcome. Immunotherapy was proposed as a new revolution in cancer treatment a few years ago, and it continues to remarkably improve the outcome of lethal malignancies. However, although results obtained with checkpoint inhibitors in mCRC were impressive, they are restricted to a small group of patients with deficient mismatch repair (dMMR) or MSI-H [110]. In this regard, distinguishing an accurate knowledge of the tumor immune microenvironment is necessary to extend more effective therapeutic strategies for overcoming tumor drug resistance. It is a remarkable aspect that, as the newest molecular characterization of CRC, CMSs are correlated with specific immune infiltration profiles under the characteristic mechanisms of immune escape [111]. In malignant status, inflammatory cells that lead to cellular immunity alterations are prompted. These cells are forced to synthesize soluble factors, such as cytokines, chemokines, and proteases, which regulate the tumor cells’ growth, differentiation, and survival. Tumor development causes considerable peritumoral inflammation that verifies the consecutive activation of pathways in the inflammatory context, stimulating sustained proliferation, resistance to apoptosis, reprogramming of the stromal environment, and genomic instability [112].

Thorsson et al. [113] have developed a new extensive immune classification of solid tumors including CRC based on gene expression profiles beyond the 10,000 patients from all 33 non-hematological TCGA cancer types. Overall, they identified six distinct immune subtypes (ISs) (C1–C6). The wound healing C1 indicated a high expression of angiogenic genes, an increased proliferation ratio, and a low Th1/Th2 proportion associated with the adaptive immune infiltration. The IFN-γ dominant C2 illustrated an elevated proliferation rate, the greatest intratumoral heterogeneity, macrophages M1/M2 polarization and CD8 T cell community, and the most significant T cell receptor (TCR) diversity. High Th17 interpreted the inflammatory C3 and Th1 genes, low to moderate proliferation, lower levels of aneuploidy, more significant somatic copy-number alterations, and the most satisfactory prognosis. The lymphocyte-depleted C4 exhibited moderate cell proliferation and intratumoral heterogeneity, as well as a dominant macrophage signature with suppressed Th1 and an elevated M2, which was associated with poor outcomes. The immunologically quiet C5 indicated the poorest lymphocyte and highest macrophage responses, affected by M2, and had low rates of proliferation and heterogeneity. Ultimately, the TGF-β dominant C6 was a small group of mixed tumors with the highest TGF-β signature and a heightened lymphocytic infiltrate with a balanced Th1/Th2 ratio. Together with C4, C6 was related to the poorest prognosis [113]. The study by Soldevilla et al. [12] examined the interplay between CMS subtyping and immune subtypes (C1–C6), as was previously described by Thorsson et al. [113]. The two major CRC ISs, C1 and C2, were present in all CMS groups, but their relative distribution varied. Indeed, the C1 wound healing IS was especially prominent in CMS2 (91%) but far less common in CMS1 tumors (46%), whereas the percentage of C1 in CMS3 and CMS4 tumors was intermediate (77–78%). Conversely, the C2 IFN-γ dominant was the most widespread IS identified in CMS1 (53%) and was diminished in CMS2 (8%). In contrast, the ratio of C2 in CMS3 and CMS4 was slightly elevated (11–13%). Other ISs were rarely present in CMS1 and CMS2. The immunological landscape of CMS3 and CMS4 was different: they were enriched in the C3 inflammatory subtype (7% and 6%, respectively), as compared with CMS1/2; CMS3 had the highest representation of the C4 lymphocyte-depleted subtype (4%), and all three cases of the TGF-β dominant phenotype pertained to the CMS4 subgroup (2.3%). No significant associations were identified with gender or tumor stage [12]. All immune subtypes demonstrated an identical number of *KRAS* mutations, except for the TGF-β dominant IS, where no *KRAS* mutations were observed. *BRAF* mutations were more frequently recognized in IFN-γ dominant (25%), lymphocyte-depleted (17%), and TGF-β dominant (33%) subtypes, although these figures are not particularly precise in these last subtypes because of the small sample size. MSI, CpG island methylator phenotype, and hypermutated status were more established in the IFN-γ dominant subtype. These outcomes revealed practical implications regarding prognosis stratification and prediction of treatment response, thus, making the immune phenotype more informative about patients’ classification. In CRC patients, the IS was found to be a predictor of survival independent of other established prognostic factors, such as age, stage at diagnosis, or primary tumor site. In contrast, the CMS classification system lost statistical significance in the Cox multivariate analysis. These findings suggest that the clinical and molecular characteristics are enriched in CMS1 [41,114]. They are also present in cases with high IFN-γ expression, and the strong immune activation observed in CMS1 may be explained by the enrichment of IFN-γ-prominent tumors in this CMS. Currently, CMS1 (MSI-like immune) patients are thought to be the most likely to benefit from immunotherapy, as this subtype includes the majority of MSI/highly mutated tumors. Nonetheless, not all CMS1 are MSI, and other CMS subgroups (i.e., CMS3) also encompass a significant portion of MSI tumors. This is related to the fact that MSI is the biomarker used in critical studies to predict the clinical effectiveness of checkpoint inhibitors in CRC and is the only available biomarker in standard clinical practice to identify immunogenic CRC. The results of Soldevilla et al. show that the immune landscape of CMS1 is distinct, an aspect that may potentially affect response to therapy [12].

Tumors defined as CMS1 molecular subtype strongly express genes implicated in T cell chemotaxes, such as *CXCL9* and *CXCL10*, genes which are necessary for homeostasis and the activation of T cells (IFN-G) and NK cells (IL-15). The chemokine CXCL13 is involved in B cell recruitment and the formation of tertiary lymphoid structures (TLS) [61]. TLS present similarities with lymph nodes; high TLS densities in CRC, especially within MSI tumors [115], along with the up-regulation of genes in the HLA I and II gene family and those connected to antigen processing and presentation, such as TAP1, TAP2, and β2-microglobulin, intimate strong anti-tumor immunity in the tumor microenvironment of the CMS1 category [61]. Despite this, CMS1 tumors may avoid immune surveillance by encoding PD-1, PDL1, CTLA-4, or LAG3 as immune checkpoint molecules [61,115].

The “canonical” subtype CMS2 with 37% distribution in all CRC cases includes CIN tumors with epithelial differentiation markers and WNT and MYC up-regulated signaling pathways. Among all four CMS groups, CMS2 displays the fewest MSI tumors [41]. In contrast to CMS1 tumors, CMS2 presents a poor intratumoral immune response defined by down-regulation of lymphocytes, monocytes, and myeloid cells. Hence, few chemokine genes involved in T cell chemotaxis and activation, or antigen genes involved in processing and presentation, are present. Furthermore, poor expression of PD-1 and PD-L1 was seen in CMS2 tumors [61,115]. The term used for the CMS2 subtype is “immune desert”; thus, the few immune cells found within these tumors are resting NK cells, naïve CD4 T cells, or B cells that are not able to mediate active anti-tumor immunity in this context [115].

The “metabolic” subtype CMS3 comprises 13% of all CRC patients. This molecular subtype consists of frequent mutations in *KRAS* and more rare cases of MSI (16% of CMS3). Metabolic dysregulation of gene expression was distinguishable in many pathways [41]. The terminology used for CMS3 as the immune landscape was “immune excluded”, and is similar to CMS2, with poor infiltration of lymphocytes, monocytes, and myeloid cells, although enriched in cells expressing PD-1, Th17 cells, naïve B and T cells, and resting T cells, indicating a dormant immune microenvironment. The expression of HLA I and class II probably differs based on the heterogeneity of tumors [61,115].

At last, CMS4 that accounts for 23% of all CRC cases, consists of tumors with an EMT, TGF-β signaling activation, and angiogenesis characteristics [41]. CMS4 has an inflammatory profile characterized by the enrichment of complement components and high levels of infiltrating lymphocytes and macrophages; hence, fewer CD8 and CD4 T cells and more Tregs than in CMS1 tumors are displayed. Furthermore, macrophages with the M2 phenotype are predominantly found in CMS4 tumors, and M1 macrophages are diminished, and, in this regard, a pro-tumoral microenvironment is developed. A strong infiltration of monocytes, eosinophils, myeloid cells, and resting DCs is seen, and levels of activated DCs and NK cells are at the lowest level [61]. The inflammatory environment introduced above supports the development of tumors via immunosuppressive and angiogenic factors, such as TGF-β, CXCL12, or VEGF, found in CMS4 tumors to a higher degree. Regardless of immunosuppressive element presentation, the expression of both HLA and immune checkpoints is retained [61,115,116]. In conclusion, the importance of generating immune responses within immune deserted or excluded tumors classified into the three CMS2–4 subtypes is explained.

CRC complexity and heterogeneity and tumor cells’ ability to escape from immune surveillance by many aspects has sparked a new horizon for developing new strategies of CRC treatment, today represented by the immune checkpoint inhibitors. The interesting aspect of personalized treatment by targeting several targets and pathways, and overcoming tumor escape mechanisms, guarantees a more successful clinical outcome for every patient in the future.

## 10. CMS and Gut Microbiome

Cancer initiation and progression result from tumor cell interconnection with their epigenetic factors and microenvironment [12]. The gut microbiome is a new area of study that has emerged to better understand the environmental impact on CRC. The gut microbiota is a complex population of bacteria, protozoa, viruses, and fungi that comprises approximately 100 trillion microbial cells. The human colon is considered a host for the microbial community. The association of gut microbiome, host immunity, and metabolism with malignancies has been noticed as a result of the advancement in high-throughput microbiome sequencing methods [117]. Intestinal dysbiosis, for instance, the enrichment of different bacterial species, namely Fusobacterium nucleatum, Peptostreptococus anaerobic, and Enterotoxigenic Bacteroides fragilis, have been found to contribute to CRC carcinogenesis. These factors have their influence by promoting tumor proliferation-inducing inflammation, affecting DNA destruction [118], and conserving tumors from immune attacks [119].

In contrast, some bacteria, such as probiotics have a potentially protective function against CRC, including Lachnospiraceae species, Bifidobacterium animalis, and Streptococcus thermophilus [119]. The accumulation of Fusobacterium nucleatum as a renowned pro-tumorigenic gut bacterium was associated with worse survival in a large cohort study by Mima et al. [120]. In addition, the gut microbiota would affect the effectiveness of chemotherapy and immunotherapy by modulating immunity. For instance, cyclophosphamide, which retains functions of either chemotherapy as an alkylating agent, or immunotherapy (by inducing an anti-tumor immune response), would cause translocation of distinct species of Gram-positive bacteria, such as Enterococcus hirae, Lactobacillus murinus, and Lactobacillus johnsoii, into secondary lymphoid organs. This translocation seemed to be vital for inducing anti-tumor pathogenic Th17 cells and memory Th1 immune response [121].

In 2017, Purcell et al. successfully examined the association between the CRC microbiome and relevant CMS subtypes to improve the understanding of the linkage between bacterial species and the molecular mechanisms underlying the CRC subtypes for the first time. A cohort of 34 CRC patients was stratified into CRC subtypes by employing data derived from RNA sequencing. Then, they inferred the pertinent abundance of bacterial taxonomic groups utilizing 16S rRNA amplicon metabarcoding. Therefore, to verify the CMS classification method, they spotted differentially expressed genes (DEGs) for each subtype and then split the DEGs into up-regulated and down-regulated genes through gene set enrichment analysis (GSEA). These authors showed that immunological marks, such as immune response, interferon gamma response, inflammatory response, TNFα signaling, and cytokine-mediated signaling, were up-regulated in CMS1 [122].

On the other hand, down-regulated DEGs in CMS1 consisted of pathways of metabolism, digestion, and morphogenesis. In CMS2, an abundance of cell cycle signatures was exhibited due to up-regulated DEGs, including the regulation of DNA damage checkpoints, cell cycle regulation, and DNA replication and synthesis. There was no enrichment of WNT-related signaling, and no significant enrichment of signaling was observed in CMS2. However, the immunological signatures were down-regulated in CMS2. In CMS3, a significant up-regulation of metabolic pathways, such as for lipid and steroid metabolism, was instead detected [122]. The 16S rRNA revealed the abundance of Fusobacteria and Bacteroidetes and diminished levels of Firmicutes and Proteobacteria in CMS1. The more comprehensive analysis of bacterial taxa utilizing non-human RNA-sequencing reads disclosed bacterial organizations attributed to the molecular subtypes. Fusobacterium hwasookii and Porphyromonas gingivalis were the most prevalent bacterial species related to the CMS1 subtype. Selenomas and Prevotella species were highly associated with CMS2, contrary to CMS3. Targeted quantitative PCR was performed by employing DNA samples derived from the CRC tumors. The results displayed the abundance of Fusobacterium nucleatum, Parvimonas micra, and Peptostreptococus stomatitis in CMS1. The CMS1 subtype has been highly associated with oral pathogens and bacterial species able to construct biofilm. Collectively, strong immunological and inflammatory marks have been correlated with the CRC subtypes [122]. A more recent study proposed onco-microbial community subtypes for CRCs based on microbiota stratification. This study further confirmed the higher abundance of Fusobacterium in CRCs classified as CMS1 [123].

Fusobacterium nucleatum retains a unique adhesin molecule called FadA, enabling it to attach and invade epithelial cells [124]. Furthermore, it contributes to colorectal carcinogenesis by recruiting infiltration immune cells [125] and modulating the E-cadherin/β-catenin signaling. Fusobacterium nucleatum has also been suggested to influence the immune response in the CRC progression and to be highly regulated by diet [126]. Fusobacterium species can arouse an immune response, particularly by recruiting T cells, and are associated with the CRC subtype distinguished by CpG island methylation, MSI, and a higher occurrence of right-sided tumors [127].

## 11. Conclusions

In summary, CMS is context specific, identifies a level of heterogeneity beyond standard genomic biomarkers, and offers a means of maximizing personalized therapy. CMS stratification is currently used in prospective studies through the integration of clinical features, genomics, transcriptomics, and microbiota to select biomarkers with high sensitivity and specificity to define optimal treatments, improving the clinical outcomes of patients. In this review article, we have discussed CMS usage in different developmental phases of CRC and preclinical models in the context of the heterogeneous biology of colon carcinomas. CMS subtyping integrates the different activities of biological programs beyond single gene mutations and can, importantly, be linked to therapy sensitivity or resistance in specific subtypes, making this an attractive method to stratify colon cancer patients. We hope that CMS implementation can yield better results for selecting treatment strategies than the current standards in clinical trials. This will ultimately facilitate moving beyond the one-size-fits-all treatment currently used and may hopefully improve disease outcomes for more colon cancer patients.

## Figures and Tables

**Table 2:** The Consensus molecular subtype characteristics.

	CMS1	CMS2	CMS3	CMS4	Unclassified/Mixed
Distribution	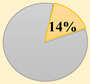	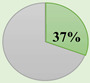	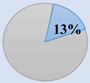	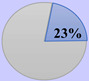	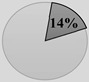
Biological features	Immune subtype MSI Tumors BRAF^V600E^ mutation Hypermutated CIMP 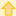 DNA damage repair genes 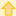	CIN 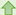 Epithelial differentiation Tumor suppressor genes 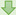 TP53 mutation	KRAS mutation 30% hypermutated CIMP 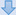	SCNA 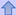 EMT 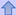	
Pathways activation	MAPK RTK JAK-STAT	WNT MYC	MAPK RTK Epithelial and metabolism signatures	TGFβ signaling Angiogenesis Matrix remodeling Stromal infiltration Complement inflammatory system	
MicroRNA		MiR-17–92 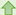	Let 7-miR family 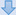	miR-200 family associated with EMT regulation 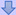	
Cell line	Undifferentiated	Colon-like	Colon-like	Undifferentiated	
Prognostic value	Good prognosis in early stage 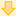 Cytotoxic lymphocytes genes overexpression Poor prognosis after recurrence	Stage 2 tumors: 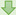 Highest OS	Stage 2 tumors: 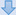 Lowest OS	Poorer prognosis	Adequate nintedanib therapy response
Predict value	Bevacizumab may be more efficient than cetuximab	Sensitivity to oxaliplatin 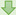 Addition of bevacizumab to capecitabine-based chemotherapy is beneficial	Addition of bevacizumab to capecitabine-based chemotherapy is beneficial 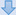	Stage III resistant to anti-EGFR therapy/no benefit from adjuvant therapy IRI-based regimen is highly beneficial Cetuximab may be more effective than Bevacizumab	
Polyps and adenomas	Same in both sexes Mostly right colon Mostly HP and SSA polyps Immune and stromal infiltration Activation of JAK-STAT and MAPK signaling BRAF^V600E^ mutation	Same in both sexes Mostly left colon Significant enrichment for WNT and MYC pathways and E2F transcription factors/G2/M checkpoint/ mitotic spindle assembly/PI3K/ mTOR genes Most of the FAP, LS, and sporadic polyps Most of AP polyps	Chang et al. study: a small proportion of polyps in Komor et al. study: metabolism-associated Most prevalent (73%) Low risk of developing CRC Enrichment in haem, fatty acid, and sugar metabolism genes	Mesenchymal and stromal signatures TGFβ activation Mostly in SSA and HP polyps Low frequency	
Immunoscore	In non-pedunculated T1 CRC: I-low			In non-pedunculated T1 CRC: I-high	
Immunotherapy and Immune Subtypes	C1 wound healing IS (46%) C2 IFN-g dominant IS (53%) Other IS rare The most likely to profit from immunotherapy	C1 wound healing IS (91%) C2 IFN-g dominant IS (8%) Other IS rare Immunity in KRAS mutant CMS2 is more suppressed	C1 wound healing IS (77–78%) C2 IFN-g dominant (11–13%) C3 inflammatory IS (7%) C4 lymphocyte-depleted IS (4%)	C1 wound healing IS (77–78%) C2 IFN-g dominant (11–13%) C3 inflammatory IS (6%) TGF-β dominant phenotype (2.3%) exclusively for CMS4	
Gut microbiome	Fusobacteria 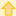 Bacteroidetes 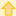 Fusobacterium Hwasookii 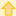 Porphyromonas Gingivalis 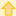 Fusobacterium nucleatum 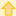 Parvimonas micra 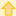 Peptostreptococcus stomatis 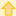 Firmicutes 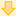 Proteobacteria 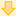	Selenomas 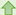 Prevotella 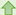 Bacteroides 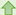	Selenomas 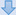 Prevotella 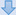		
